# The Effectiveness of Pan-Sharpening Algorithms on Different Land Cover Types in GeoEye-1 Satellite Images

**DOI:** 10.3390/jimaging9050093

**Published:** 2023-04-30

**Authors:** Emanuele Alcaras, Claudio Parente

**Affiliations:** DIST—Department of Science and Technology, Parthenope University of Naples, Centro Direzionale, Isola C4, 80143 Naples, Italy

**Keywords:** data fusion, pan-sharpening algorithms, VHR, GeoEye-1, land cover, quality assessment

## Abstract

In recent years, the demand for very high geometric resolution satellite images has increased significantly. The pan-sharpening techniques, which are part of the data fusion techniques, enable the increase in the geometric resolution of multispectral images using panchromatic imagery of the same scene. However, it is not trivial to choose a suitable pan-sharpening algorithm: there are several, but none of these is universally recognized as the best for any type of sensor, in addition to the fact that they can provide different results with regard to the investigated scene. This article focuses on the latter aspect: analyzing pan-sharpening algorithms in relation to different land covers. A dataset of GeoEye-1 images is selected from which four study areas (frames) are extracted: one natural, one rural, one urban and one semi-urban. The type of study area is determined considering the quantity of vegetation included in it based on the normalized difference vegetation index (NDVI). Nine pan-sharpening methods are applied to each frame and the resulting pan-sharpened images are compared by means of spectral and spatial quality indicators. Multicriteria analysis permits to define the best performing method related to each specific area as well as the most suitable one, considering the co-presence of different land covers in the analyzed scene. Brovey transformation fast supplies the best results among the methods analyzed in this study.

## 1. Introduction

In order to increase the accuracy of a measurement, it is sometimes possible to merge data obtained from different sensors [1]. These techniques allow to achieve improved accuracies compared to those that could be obtained using only a single sensor and are called data fusion techniques [2].

Data fusion techniques applied in the field of remote sensing allow the integration of data from multiple sensors and hence the production of more coherent, accurate and useful information than that which is provided by each single sensor [3]. Additionally, they find increasing diffusion in the related technical scientific fields, such as Earth observations and the accurate monitoring of the dynamics affecting the territory and the environment [4]. Such techniques become fundamental for many studies and applications, concerning, for example, the effects of climate change [5], desertification processes [6], deforestation [7], coastal erosion [8], burned area recognition [9], the phases of urban development [10], seismic damage evaluation [11] and cultural heritage preservation [12]. In fact, it is undeniable that the information from certain types of sensors remains partial, while the integration of heterogeneous data allows us to highlight specificities and details that would not be detected by sectoral analysis [13]. Nevertheless, data acquired from optical sensors can be integrated with the data of a different nature, such as LiDAR [14], SAR [15] and microwave [16], which are useful for better object identification and classification.

Among the data fusion techniques, those relating to pan-sharpening play a particularly important role, thanks to which, it becomes possible to merge the high geometric resolution of panchromatic images with the high spectral resolution of the multispectral bands [17]. As it is known, to reduce the effects of noise on signals [18], the sensors that acquire information in the panchromatic band, operating in a relatively broader band (including the entire visible range, sometimes even the near infrared) allow high geometric resolutions to be reached, in certainly better conditions than those obtainable in the case of multispectral bands. In fact, as the bandwidth decreases, it becomes necessary in order to optimize the signal-to-noise ratio to increase the size of the ground resolution cell; it follows that the multispectral images have a lower geometric resolution than the one that characterizes the panchromatic data [19,20]. The pan-sharpening techniques allow us to overcome this limit, so multispectral data with the same pixel size of the panchromatic data are achieved. The algorithms present in the literature which are useful for this purpose are many in quantity, and the results that can be obtained from their application are different: as a consequence, a careful evaluation process of the characteristics of the images derived from the pan-sharpening function applications is required [21]. In 2015 Vivone et al. [22] provided an effective analysis and comparison of the classic pan-sharpening methods belonging to the component substitution or multiresolution analysis families.

The pan-sharpened images provide the highest level of detailed information and are also useful to support the multi-representation of geographical data [23]. For this reason, pan-sharpening studies have had a great improvement in recent years: an edge-adaptive pan-sharpening method was proposed in Rahamani et al.’s work to enforce spectral fidelity away from the edges [24]; and more recently, Masi et al. proposed to use convolutional neural networks (CNN) for pan-sharpening [25]. Ultimately, deep learning techniques were investigated by several researchers. In some cases, they start from and adapt super-resolution (SR) [26,27,28], a technique that enhances minute details of the features in an image, thereby improving the image’s spatial information. Rohith and Kumar [29] tested and analyzed ten state-of-the-art SR techniques based on deep learning techniques using ten different publicly available datasets; in addition, they proposed a new method that is based on the integration of SR with a band-dependent spatial detail (BDSD) algorithm [30]. Xiong et al. [31] designed a loss function suitable for pan-sharpening and a four-layer convolutional neural network capable of adequately extracting spectral and spatial features from the original source images; the approach does not require the designed loss function to have the reference fused image so as to avoid the preprocessing of the data and generate training samples. Jones et al. [32] introduced the normalized difference vegetation index in the CNN, so the spectral distortions produced by pan-sharpening were reduced through taking the normalized difference ratio of the spectral bands. In 2020, Vivone et al. [33] revisited pan-sharpening with classical and emerging pan-sharpening methods, but mainly focused on non-deep learning methods, while in 2022, Deng et al. [34] provided a more detailed comparison for deep learning methods.

The most recent studies subsequently turn their attention to both deep learning approaches and land covers. In fact, pan-sharpening is also applied for land cover mapping [35,36] and vice versa; pan-sharpening methods are often tested for different land covers [37]. A pan-sharpening algorithm can provide different results in relation to the investigated scene, so it became of fundamental importance to test and find the most performing methods [38].

This paper focuses on the latter aspect: to analyze pan-sharpening techniques in relation to different land covers. A GeoEye-1 dataset is taken into analysis, from which four different areas are extracted. Each area, that we call a frame, presents a different land cover and is classified into urban, semi-urban, rural or natural, depending on the quantity of vegetation it presents. For each frame, nine pan-sharpening techniques (intensity-hue-saturation, intensity-hue-saturation fast, Brovey transformation, Brovey transformation fast, Gram–Schmidt mode 1, Gram–Schmidt fast, Gram–Schmidt mode 2, smoothing filter intensity-based modulation, high-pass filter) are applied and compared to find the most performing one in consideration of spectral similarity with the original multispectral images and spatial correlation with the panchromatic one. In order to assess the reliability of the methods, four indices are taken into account, particularly two spectral indices (UIQI and ERGAS) and two spatial indices (Zhou index and spatial ERGAS). Finally, a multicriteria analysis is carried out to find the best performing algorithm for each type of frame as well as the most suitable one, considering the co-presence of different land covers in the analyzed scene.

All the operations are carried out using software free and open source, i.e., Quantum GIS version 3.10.3 [39] and SAGA GIS 2.3.2 [40].

## 2. Materials and Methods

### 2.1. Dataset and Study Areas

For this paper, a GeoEye-1 dataset is chosen. Formerly known as OrbView-5, GeoEye-1 satellite was launched on 6 September 2008, as a next-generation high-resolution imaging mission. GeoEye-1 images are used in a wide variety of applications, such as cartography and location-based services, risk management, environmental monitoring and natural resources, defense and national security [41].

GeoEye-1 imaging system is a push-broom imaging system, which supplies panchromatic (PAN) and multispectral (MS) images, as reported in Table 1 [42].

**Figure 1:**
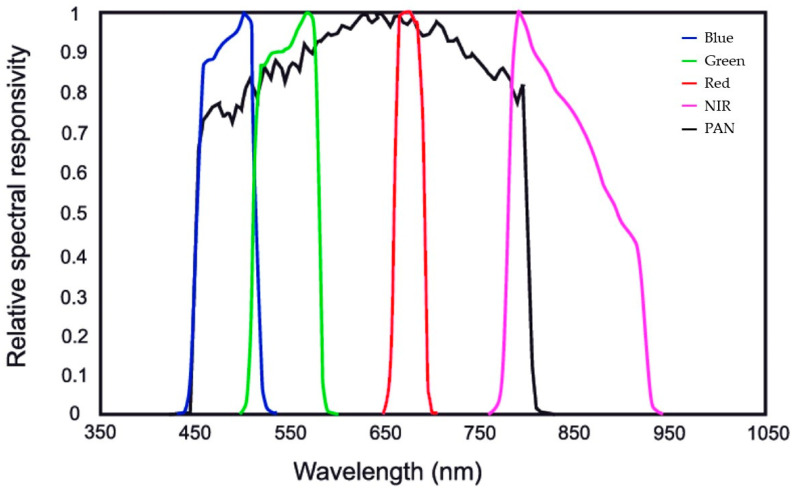
Spectral response of GeoEye-1 MS and PAN sensors as stated by the European Space Agency [42].

The spectral response associated with the GeoEye-1 MS and PAN sensors is shown in Figure 1.

**Figure 2:**
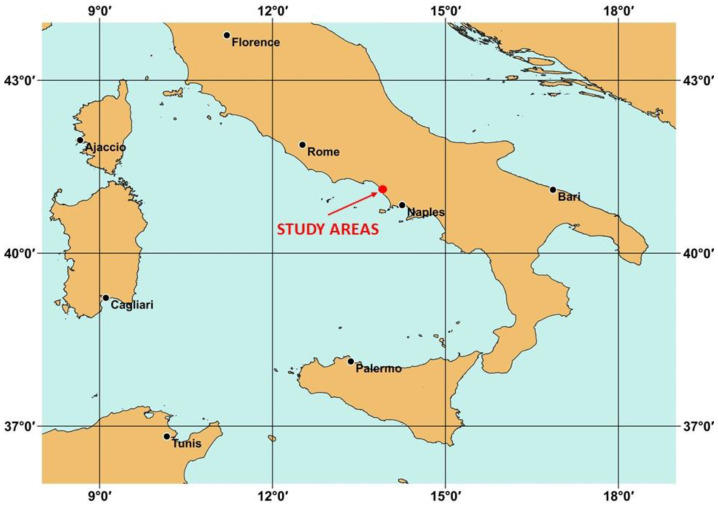
The geolocalization of the study areas in Italy.

The chosen dataset is localized in the Campania region (Italy), as shown in Figure 2.

**Figure 3:**
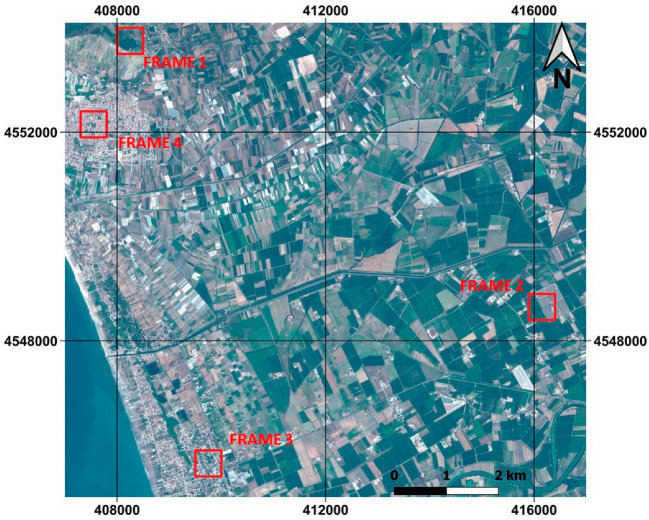
Location of the study areas and the GeoEye-1 dataset.

**Figure 4:**
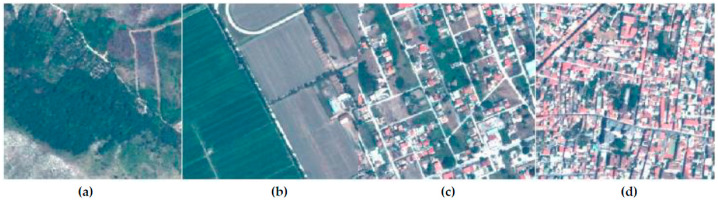
The study areas, from the left: frame 1—(**a**) natural area; frame 2—(**b**) rural area; frame 3—(**c**) semi-urban area; frame 4—(**d**) urban area.

Particularly, it concerns the stretch of land extending from the mouth of the river Volturno in the south, to the city of Mondragone in the north. The images extend from the coastal zone in the west to the inland areas in the east, which are rich in crops. Varied land covers are therefore present, passing from urban to rural environments. The dataset is georeferenced in UTM/WGS84 (Zone 33 N) coordinate system. The 4 study areas chosen for this article are extracted from the same Geoeye-1 imagery as shown in Figure 3 and in detail in Figure 4.

The frames chosen for this study have an area of 0.25 Km^2^ each (500 m × 500 m), and can be described as follows:

- Frame 1—A natural area, it presents a forest (green area) and nude soil, no man-made features (included between coordinates East 408,000 m–408,500 m and North 4,553,500 m–4,554,000 m);
- Frame 2—A rural area, it mainly presents two kinds of cultivated areas, prevalently covered by vegetation, slightly man-made (included between coordinates East 415,900 m–416,400 m and North 4,548,400 m–4,548,900 m);
- Frame 3—A semi-urban area, it presents a mix of land cover, such as houses, vegetation and nude soil, averagely man-made (included between coordinates East 409,500 m–410,000 m and North 4,545,400 m–4,545,900 m);
- Frame 4—An urban area, it presents few green areas, mostly represented by trees, and a typical urban land cover with houses, strongly man-made (included between coordinates East 407,300 m–407,800 m and North 4,551,900 m–4,552,400 m).

In this way, according to the approach proposed by Meng et al. [43], we have samples that are emblematic for the typical thematic surface features present in the study area.

### 2.2. Classification

The study areas are classified into urban, semi-urban, rural and natural based on the quantity of vegetation present in them. For this purpose, the normalized difference vegetation index (NDVI) is applied, the formula of which is expressed as follows [44]:

(1) $$NDVI=\frac{NIR-RED}{NIR+RED}$$

NDVI highlights the vegetated areas with respect to the bare soil, so that the vegetation is represented with higher brightness values than the rest.

The classification is therefore carried out by applying the maximum likelihood classification (MLC), a supervised classification technique [45] employing training sites to estimate statistical characteristics of the classes, which are used to evaluate probabilities that a pixel is assigned to a determinate class [46]. MLC is applied directly on NDVI.

### 2.3. Pan-Sharpening Methods

In the literature, there are many pan-sharpening techniques useful for fusing the high spectral resolution of MS with the high spatial resolution of PAN [47,48]. The following 9 algorithms are applied in this study: intensity-hue-saturation (IHS), IHS fast (IHSF), Brovey transformation (BT), Brovey transformation fast (BTF), Gram–Schmidt mode 1 (GS1), Gram–Schmidt fast (GSF), Gram–Schmidt mode 2 (GS2), smoothing filter intensity-based modulation (SFIM) and high-pass filter (HPF). The characteristics of the methods are reported below.

#### 2.3.1. Intensity-Hue-Saturation

The IHS method is based on switching from the RGB (red-green-blue) to the IHS (intensity-hue-saturation) color model [49]. The intensity component, which is a synthetic panchromatic image (S), is used to fuse PAN and MS data according to the fusion framework, called the generalized IHS (GIHS) [50], where the intensity component is supplied by:

(2) $$S=\frac{1}{n}\sum_{k=1}^{n}{MS}_{k}$$

where *n* represents the number of the multispectral bands and ${MS}_{k}$ is the k-th multispectral image.

The pan-sharpened multispectral images are produced using the following formula:

(3) $${MS}_{k}^{\prime }={MS}_{k}+PAN-S$$

where ${MS}_{k}^{\prime }$ is the k-th pan-sharpened image.

By analyzing the spectral response of the original dataset, weights can be introduced to calculate S [51]. This is the so-called IHS fast *(IHSF)*, where S is obtained as follows [52]:

(4) $$S=\frac{1}{\sum_{1}^{n}w_{k}}\sum_{k=1}^{n}w_{k}\cdot {MS}_{k}$$

where $w_{k}$ is the weight of k-th multispectral band.

#### 2.3.2. Brovey Transformation

The Brovey transformation (BT) was developed to visually increase the contrast in the low and high ends of an image’s histogram and thus change the original scene’s radiometry [53]. The BT pan-sharpened image can be computed as [54]:

(5) $${MS}_{k}^{\prime }=\frac{{MS}_{k}}{S}\cdot PAN$$

where S is the synthetic panchromatic image.

As for IHS, when weights are introduced to calculate S, this approach is called Brovey transformation fast (BTF).

#### 2.3.3. Gram–Schmidt Transformation

The Gram–Schmidt pan-sharpening method is based on the mathematical approach of the same name, by applying the orthonormalization of a set of vectors; particularly, in the case of images, each band (panchromatic or multispectral) corresponds to one vector [55]. To apply the Gram–Schmidt transformation (GST), the first step is to create a lower resolution panchromatic image from the multispectral band images (S). GST is performed to orthogonalize and decorrelate S and the MS bands. Particularly, S is used as the first band in the Gram–Schmidt process. At the end of the transformation, the PAN takes the place of S and the inverse GST is performed to produce the enhanced spatial resolution multispectral digital image [56]. The fused bands are obtained as follows:

(6) $${MS}_{k}^{\prime }={MS}_{k}+g_{k}(PAN-S)$$

where $g_{k}$ is the gain, given by:

(7) $$g_{k}=\frac{cov\left({MS}_{k},S\right)}{var(S)}$$

where $cov\left({MS}_{k},S\right)$ is the covariance between the initial k-th multispectral image and the synthetic image; var(S) is S variance.

Different versions of GST are available, depending on the way S is generated. The simplest way to produce the synthetic image is supplied by Equation (2): in this case, the method is named GS mode 1 (GS1). If weights are introduced, this method is referred as Gram–Schmidt fast (GSF) [57].

Another possibility is to degrade the panchromatic by applying a smoothing filter. The degraded image (D) is then used as follows:

(8) $${MS}_{k}^{\prime }={MS}_{k}+g_{k}\left(PAN-D\right)$$

This method is known as Gram–Schmidt mode 2 (GS2).

#### 2.3.4. Smoothing Filter-Based Intensity Modulation

This technique was developed by Liu and is based on the concept that, by using a ratio between a PAN and its low-pass filtered image (D), spatial details can be modulated to a co-registered lower resolution multispectral image without altering its spectral properties and contrast [58].

In this case, the gains can be considered as:

(9) $$g_{k}=\frac{{MS}_{k}}{D}$$

The fused images are produced as follows:

(10) $${MS}_{k}^{\prime }={MS}_{k}+\frac{{MS}_{k}}{D}\left(PAN-D\right)$$

#### 2.3.5. High-Pass Filter

The high-pass filter method (HPF) was introduced by Chavez and Bowel [59]. According to Vivone et al., the high frequency component of the PAN image can be extracted by applying the smoothing filter to the PAN image and subtracting the result to the PAN as follows [22]:

(11) $${MS}_{k}^{\prime }={MS}_{k}+PAN-D$$

### 2.4. Quality Assessment

To evaluate the quality of the pan-sharpened data, various indices are available in the literature [60], particularly those investigating the spectral correlation with the MS images and the spatial similarity with PAN [61]. In this paper, universal image quality index (UIQI) and erreur relative globale adimensionalle de synthèse (ERGAS) are adopted as indicators of the spectral correlation between the MS original bands and the fused ones; Zhou index (ZI) and spatial ERGAS (S-ERGAS) are used to determine the spatial similarity between PAN and the fused images. The adopted indices are briefly reported below.

#### 2.4.1. Universal Image Quality Index (UIQI)

This index takes into account three components and is obtained as follows [62]:

(12) UIQI=cov(MSk,MSk′)varMSkvar(MSk′)·2(MSk)(MSk′)(MSk)2+(MSk′)2·2varMSkvar(MSk′)varMSk+var(MSk′)

where $cov\left({MS}_{k},{MS}_{k}^{\prime }\right)$ is the covariance between the initial k-th multispectral image and the corresponding pan-sharpened image; $var\left({MS}_{k}\right)$ is ${MS}_{k}$ variance; $var({MS}_{k}^{\prime })$ is ${MS}_{k}^{\prime }$ variance; (MSk) is the mean value of ${MS}_{k}$ and (MSk′) is the mean value of ${MS}_{k}^{\prime }$.

The range of *UIQI* is [−1, 1]: values close to 1 indicate a good performance of the pan-sharpening technique [63].

#### 2.4.2. Erreur Relative Globale Adimensionalle de Synthèse (ERGAS)

It quantifies the spectral quality of the fused images with the following formula [64]:

(13) $$ERGAS=100\cdot \frac{h}{l}\cdot \sqrt{\frac{1}{n}\cdot \sum_{k=1}^{n}{\left(\frac{RMSE({MS}_{k})}{\mu_{k}}\right)}^{2}}$$

where *h* is the spatial resolution of reference image (PAN); *l* is the spatial resolution of original multispectral images (${MS}_{k}$); *n* is the number of spectral bands and *µ_k_* is the mean of the k-th band of the original image. RMSE is the root mean square error for k-band between fused (${MS}_{k}^{\prime }$) and original bands (${MS}_{k}$) and is obtained as follows [65]:

(14) $$RMSE=\sqrt{\frac{1}{MN}\sum_{i=1}^{M}\sum_{j=1}^{N}{\left({MS}_{k}\left(i,j\right)-{MS}_{k}^{\prime }\left(i,j\right)\right)}^{2}}$$

where ${MS}_{k}\left(i,j\right)$ represents the pixel value in the original (reference) image; ${MS}_{k}^{\prime }\left(i,j\right)$ is the pixel value in the corresponding fused image; i and j identify the pixel position in each image and M and N are, respectively, the number of rows and the number of columns that are present in each image. Low values of ERGAS suggest a likeness between original and fused bands.

#### 2.4.3. Zhou’s Spatial Index (ZI)

As a first step, the high frequency information from PAN and ${MS}_{k}^{\prime }$ is extracted by using a high frequency Laplacian filter:

(15) $$Laplacian\;Kernel=\left[\begin{array}{ccc} -1 & -1 & -1\\ -1 & 8 & -1\\ -1 & -1 & -1 \end{array}\right]$$

As a result, the high-pass PAN (HPPAN) and the high-pass ${MS}_{k}^{\prime }$ ${(HPMS}_{k}^{\prime })$ are obtained and used to calculate ZI as follows [66]:

(16) $$ZI=\frac{cov(HPPAN,{HPMS}_{k}^{\prime })}{\sqrt{var\left(HPPAN\right)var({HPMS}_{k}^{\prime })}}$$

#### 2.4.4. Spatial ERGAS (S-ERGAS)

By introducing a spatial RMSE, it is possible to redefine ERGAS as a spatial index [67]. Spatial RMSE is achieved as follows:

(17) $$Spatial\;RMSE=\sqrt{\frac{1}{MN}\sum_{i=1}^{M}\sum_{j=1}^{N}{\left(PAN-{MS}_{k}^{\prime }\left(i,j\right)\right)}^{2}}$$

## 3. Results and Discussion

### 3.1. Classification Results

**Figure 5:**
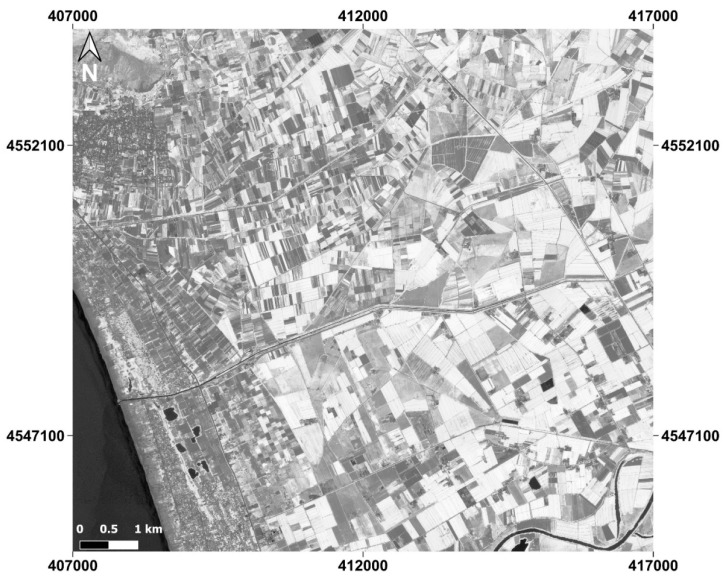
NDVI applied to the GeoEye-1 dataset.

The application of NDVI generates a synthetic band, as shown in Figure 5.

**Figure 6:**
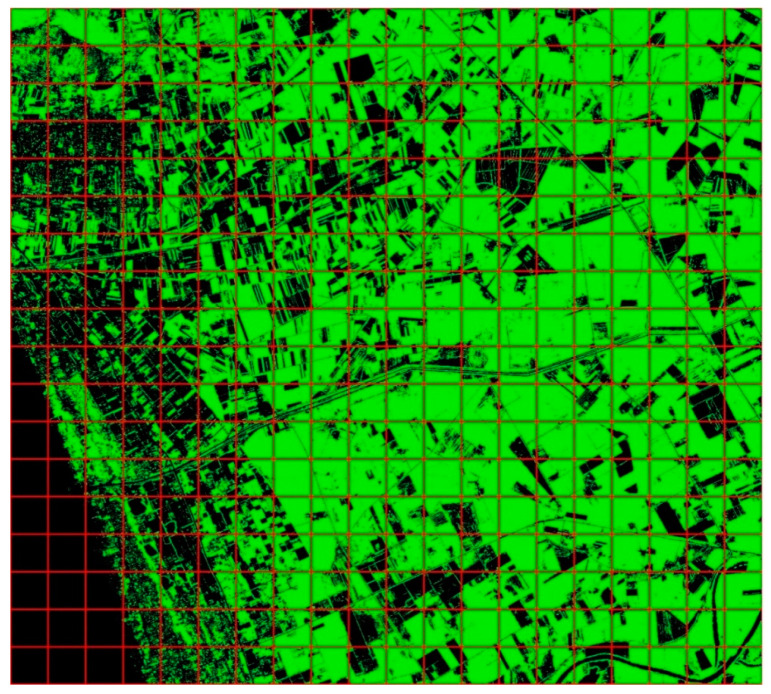
The result of the classification: green pixels identify vegetation.

By applying the MLC, two classes (vegetation/non-vegetation) are identified, and a regular grid of half a kilometer is applied to the classified dataset, as shown in Figure 6.

The four categories are identified based on the percentage of vegetation pixels according to the following thresholds:

- Natural area: 75–100%;
- Rural area: 50–75%;
- Semi-urban area: 25–50%;
- Urban area: 0–25%.

**Figure 7:**
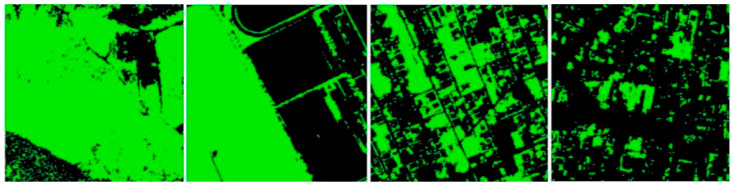
The classified areas, from the left: natural area (80.67% of vegetation); rural area (52.44% of vegetation); semi-urban area (41.16% of vegetation); urban area (17.59% of vegetation).

Figure 7 shows the results of the classification on the four selected frames.

### 3.2. Pan-Sharpening Results

Table 2 shows the results of the fusions for frame 1.

In frame 1, the HPF method presents the best results in terms of spectral correlation with the original images, since it provides the higher values of UIQI and the lowest ERGAS. Good results are also provided by SFIM, BTF and IHSF. The UIQI values vary significantly between bands in each method. In terms of spatial similarity, BT, BTF, IHSF and GS1 present the best results. Typically, a band that provides the higher UIQI has the lowest ZI and vice versa, as can be seen, for example, in HPF, GS2, GSF and IHSF. Overall, we can declare that BTF and IHSF are the most performing methods in frame 1.

Table 3 shows the results of the fusions for frame 2.

In frame 2 HPF, SFIM and GS2 provides the best results in terms of spectral correlation with the original images, in particular, HPF is the most performing one in terms both of UIQI and ERGAS. BT and BTF show the greatest spatial similarity, although S-ERGAS values do not have not very good variabilities for each method. GS1 presents a particular case since it provides the lowest S-ERGAS values, but also the lowest ZI; this demonstrates the importance of taking into account different indices. Overall, it can be said that HPF, SFIM and GS2 are the most performing methods for frame 2. This area is characterized by a lower variability in terms of features if compared to frame 1; particularly, it presents two uniformly cultivated zones that supplied the highest values of UIQI, because in this case, pan-sharpening application does not introduce a high level of shape enhancement and similarity between original MS image and fusion products. This effect is more evident in the NIR band due to the highest reflectance in the presence of both soil and vegetation [68].

Table 4 shows the results of the fusions for frame 3.

In frame 3, HPF and GSF are the best methods in terms of spectral correlation, followed by IHSF and BTF. Exceptional results are presented by BT in terms of spatial similarity, but BTF, IHSF and GSF also provide good results. SFIM, HPF and GS2 present the worst results in spatial similarity due to the relatively high variability of the images if compared with frames 1 and 2: these methods use a low-pass filter to degrade the PAN, so the boarders of the features are less defined [69]. Overall, BTF, IHSF and GSF are the most performing methods in frame 3.

Table 5 shows the results of the fusions for frame 4.

In frame 4, IHSF and HPF are the best methods in terms of spatial correlation. Additionally, GSF and BTF provide good results. BT is the most performing technique in terms of spatial similarity, followed by BTF, IHSF and GSF. Overall, IHSF is the best method in frame 4. This area presents the greatest variability in terms of features, so the methods that apply the low-pass filter (HPF, SFIM and GS2) do not perform well in spatial terms. Frame 4 can be seen as an opposite situation with respect to frame 2: the first is a completely urbanized area, including mostly buildings, roads and few trees, while the second includes two very large homogeneous cultivated areas with very few variations.

As already stated in other studies, comparing each frame, it is not possible to choose a pan-sharpening technique a priori, since each method performs in a different way in relation to the land cover [70,71].

What emerges from this study can be summarized as follows:

- Weighted methods always perform better than the respective unweighted techniques in terms of spectral correlation;
- Weighted methods tend to maintain the ZI and S-ERGAS values of their respective unweighted methods;
- Low-pass filter-based techniques perform quite well in low-variating land covers, but tend to perform poorly in variegated land cover;
- Low-pass filter-based techniques never present the best performance in terms of spatial similarity with PAN.

As already stated in other studies [72], usually, when pan-sharpening is applied, the better the image spatial quality, the worse the image spectral quality and vice versa. In order to find a compromise in this paper, we apply the multicriteria analysis [73] approach proposed by Alcaras et al. in 2021 [74]:

- A ranking is made for the methods in consideration of each indicator, assigning a score from 1 to 9.
- The spectral indicators are then mediated between them, as well as the spatial indicators.
- A general ranking is obtained by averaging the two results.

Finally, the general ranking of the methods for each frame is shown in Table 6, where rank 1 is assigned to the method in the first position, rank 2 to the method in the second position, and so on.

**Figure 8:**
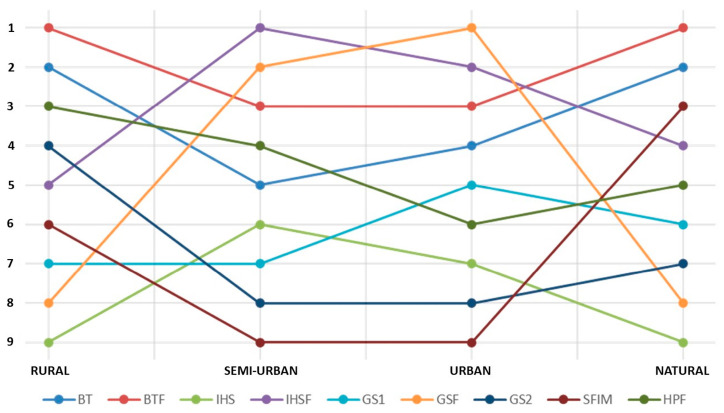
Trends of the pan-sharpening algorithms in each frame.

To better understand the performances of each method, Figure 8 shows the trend of the pan-sharpening algorithms in each frame.

The results show excellent performances of the BTF algorithm in the most vegetated areas, i.e., rural and natural areas. In the semi-urban area, the best results were achieved by the exploitation of the IHSF, while in the urban area, the most efficient algorithm is the GSF. In general, the “fast” methods are the most reliable, especially the BTF, which is the only method to consistently be in the top three of the best methods in all frames. The IHS method does not present good results in areas where the vegetation is higher than 50% (rural and natural), and on the contrary, the SFIM does not present good results in areas with a low vegetation rate, i.e., below 50% (semi-urban and urban).

Finally, we would like to underline that each of the indicators used manages to highlight the level of spectral or spatial quality of the pan-sharpened image. There are no studies in the literature that identify an indicator as the most performing; for this reason, four indicators (i.e., UIQI, ERGAS, ZI, S-ERGAS) are considered in our experiments. The multicriteria analysis approach adopted is believed to strike the right balance by bringing the different indicators into play.

**Figure 9:**
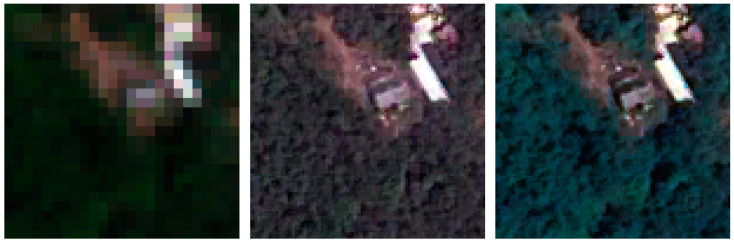
A detail of frame 1: on the left, the RGB composition of the MS images; in the middle, the RGB composition of IHS pan-sharpening; on the right, the RGB composition of BTF pan-sharpening.

**Figure 10:**
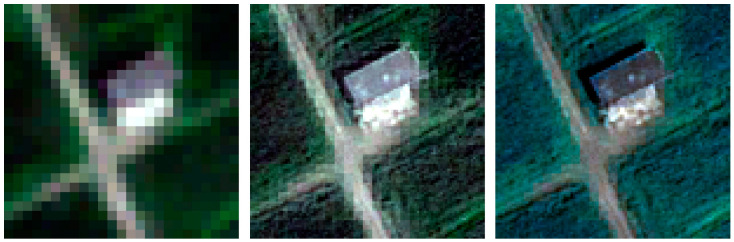
A detail of frame 2: on the left, the RGB composition of the MS images; in the middle, the RGB composition of IHS pan-sharpening; on the right, the RGB composition of BTF pan-sharpening.

**Figure 11:**
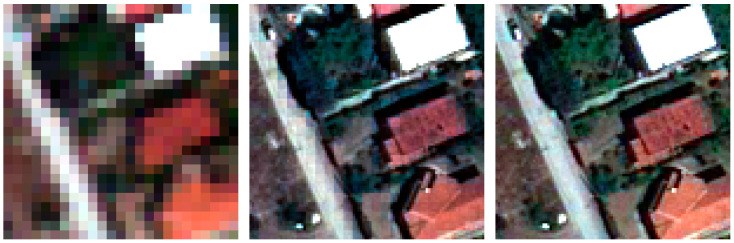
A detail of frame 3: on the left, the RGB composition of the MS images; in the middle, the RGB composition of SFIM pan-sharpening; on the right, the RGB composition of IHSF pan-sharpening.

**Figure 12:**
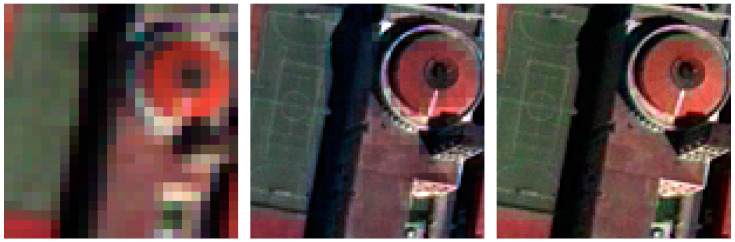
A detail of frame 14: on the left, the RGB composition of the MS images; in the middle, the RGB composition of SFIM pan-sharpening; on the right, the RGB composition of GSF pan-sharpening.

According to the concept, a visual inspection of the resulting images is useful to evaluate the color preservation quality and the spatial improvements in object representation [75]. Figure 9, Figure 10, Figure 11 and Figure 12 show the RGB composition of the multispectral pan-sharpened images obtained for the least and best-performing methods in each frame (according to Table 6) compared with the corresponding initial RGB image composition.

By means of visual inspection, it is evident that, in general, the fusion process leads to an enhancement of the geometric resolution.

From the analysis of frame 1, it is evident that the application of the IHS algorithm (the least performing) generates darker colors and a yellowing of the RGB image, and the enhanced geometric resolution is, however, appreciable. The result produced by applying the BTF method (the best in this frame) instead generates an RGB image with colors closer to those of the initial RGB composition.

The geometries of the second frame are different compared to the first one, but the scene still shows a high vegetated area. The IHS method (in this case, the worst one) again generates darkened images and presents some areas in which the grid typical of the MS image is visible. The grid completely disappears in the RGB image produced using the BTF method (the best in this frame), which presents, in its RGB composition, brighter colors.

The detail of frame 3 shows a reduced presence of vegetation compared to the first two frames. In the RGB composition of the SFIM method (in this case, the least performing), the pixels that form the road present the typical grid of the MS image, while adequately preserving the color of the scene. On the other hand, the square pattern is not visible in the RGB composition of the IHSF method (the most performing of frame 3).

The detail relating to frame 4 presents a football field and the roof of a church: the results obtained are satisfactory; as can be seen, the lines of the football field are clearly visible in the pan-sharpened images, which is not possible in the RGB composition of the initial images. The SFIM method (the least performing in this frame) presents the grid typical of MS images along the edges of the roof of the church, which, on the opposite, cannot be found in the RGB composition of the GSF method (the best in this frame). However, the dome of the church presents the peculiar square pattern in both the SFIM method and the GSF method.

## 4. Limitations and Future Research Directions

The methods analyzed in this article fall within the classical approaches to pan-sharpening and highlight how the performances are different in relation to the type of land cover. However, the limitations of the approach are evident, and we can identify at least three lacks.

Firstly, the analysis is conducted for a single type of image, GeoEye-1: even if the applications could be applied in relation to other types of sensors operating in the same bands (such as IKONOS and Pléiades), the ranges of acquisition wavelengths may be slightly different. Consequently, the results should be properly analyzed and evaluated for each sensor type products.

Secondly, the methods are applied on four frames, chosen according to the amount of vegetation present in each of them. The analysis of the results would be much more robust if the methods are applied to a greater number of samples.

Finally, the pan-sharpening techniques analyzed are among the classics, not considering the new pan-sharpening trends that generally apply deep learning and neural networks techniques.

In order to overcome the three limitations identified above, we will conduct a larger study in the future that will cover more types of high-resolution satellite images, analyze a greater number of samples, and also include the most recent pan-sharpening trends. Particularly, approaches to synthesize high-resolution MS based on deep learning and CNN will be considered, analyzing and comparing innovative methods such as those proposed by Jeong and Kim [76], Xu et al. [77] and Liu et al. [78].

## 5. Conclusions

In this paper, nine pan-sharpening techniques (IHS, IHSF, BT, BTF, GS1, GSF, GS2, SFIM, HPF) are compared in relation to four specific areas. Each area has different land covers and responds in a distinct way to the pan-sharpening applications. Particularly, the following representative zones are considered: natural area, rural area, semi-urban area and urban area. The frames are selected from a GeoEye-1 dataset localized in the Campania region (Italy), north of Naples.

The classification of each area takes place on the basis of the amount of vegetation present in practice by evaluating the percentage of pixels classified as vegetation, following the application of NDVI and MLC.

To evaluate the performance of each method, visual, spectral and spatial analyses are carried out. Visual analysis is performed considering RGB true color composition, while spectral and spatial analyses are based on quality indices calculation: UIQI and ERGAS for spectral inspection and ZI and S-ERGAS for spatial similarity evaluation.

Each quality index provides a different indication, and for this reason, a multicriteria analysis is carried out to identify the best algorithm in each area.

The performance of each pan-sharpening method is different in relation to the considered frame. However, each selected frame does not supply the same ranking of the method performances.

The introduction of the weights to define the synthetic panchromatic image in some methods (BTF, IHSF, GSF) allows us to enhance the resulting performance in terms of spectral correlation, and to maintain the spatial similarity to the panchromatic image ensured by the respective unweighted methods (BT, IHS, GS).

Pan-sharpening methods based on low-pass filter-based application (GS2, SFIM, HPF) do not provide optimal results for the selected frames. However, SFIM supplies good performances for the natural areas, which present a homogeneous land cover while presenting poor results for variegated land cover, i.e., urban and semi-urban areas. Similar poor results are achieved by the GS2 algorithm in urban and semi-urban areas. HPF trends are in line with the previous two methods, but generally provides better performances. Particularly, the similarity of the resulting fused products with PAN image is low.

The results of this study suggest that, in relation to GeoEye-1 images, the best algorithms for pan-sharpening are: BTF for rural areas, as well as for natural areas; IHSF for semiurban areas; and GSF for urban areas. When the analyzed scenes show the co-presence of different types of areas, the most effective method is BTF as it is able to provide acceptable results even when it is not the most performing one. Considering the variability of the areas that may occur as well as the specificity of the used sensors, i.e., acquisition bands and spectral response, a comparison of different pan-sharpening methods is recommended and the multicriteria approach adopted in this article is useful to select the most performing one.

To include all of the abovementioned considerations in the pan-sharpening process, an automation of the comparison of different approaches is suggested to facilitate and support the user to select the most performing algorithm. To reduce the processing and calculation time, a first selection of the algorithms to be compared can be performed by taking into account the results of this study; in fact, our experiments already highlight the performance among some of the most efficient and widespread methods.

Finally, future work will investigate convolutional neural network algorithms based on deep learning to implement pan-sharpening in high and very high resolution images.

## Figures and Tables

**Table 1:** Characteristics of GeoEye-1 images.

Bands	Wavelength (nm)	Resolution (m)
Panchromatic	450–800	0.5
Band 1—Blue	450–510	2
Band 2—Green	510–580	2
Band 3—Red	655–690	2
Band 4—Near Infrared (NIR)	780–920	2

**Table 2:** Pan-sharpening quality indices values for frame 1.

Method	Bands	UIQI	UIQI_M_	ERGAS	ZI	ZI_M_	S-ERGAS
BT	Blue	0.812	0.842	7.458	0.950	0.923	6.250
	Green	0.885			0.964		
	Red	0.929			0.849		
	NIR	0.743			0.930		
BTF	Blue	0.656	0.820	4.280	0.978	0.928	6.551
	Green	0.829			0.990		
	Red	0.953			0.907		
	NIR	0.842			0.838		
IHS	Blue	0.783	0.794	9.952	0.922	0.860	6.717
	Green	0.746			0.947		
	Red	0.708			0.905		
	NIR	0.937			0.664		
IHSF	Blue	0.758	0.849	4.390	0.983	0.882	6.674
	Green	0.789			0.990		
	Red	0.894			0.975		
	NIR	0.954			0.578		
GS1	Blue	0.904	0.825	9.553	0.914	0.899	6.672
	Green	0.836			0.946		
	Red	0.676			0.908		
	NIR	0.884			0.826		
GSF	Blue	0.827	0.860	4.981	0.983	0.777	6.748
	Green	0.827			0.989		
	Red	0.801			0.986		
	NIR	0.984			0.149		
GS2	Blue	0.861	0.877	4.841	0.956	0.860	7.363
	Green	0.840			0.960		
	Red	0.848			0.956		
	NIR	0.957			0.568		
SFIM	Blue	0.745	0.851	4.018	0.963	0.896	7.059
	Green	0.865			0.946		
	Red	0.965			0.823		
	NIR	0.828			0.852		
HPF	Blue	0.845	0.898	3.797	0.959	0.851	7.085
	Green	0.859			0.956		
	Red	0.931			0.915		
	NIR	0.956			0.574		

**Table 3:** Pan-sharpening quality indices values for frame 2.

Method	Bands	UIQI	UIQI_M_	ERGAS	ZI	ZI_M_	S-ERGAS
BT	Blue	0.895	0.891	7.215	0.939	0.905	4.975
	Green	0.914			0.947		
	Red	0.943			0.833		
	NIR	0.812			0.903		
BTF	Blue	0.664	0.837	4.226	0.979	0.917	5.152
	Green	0.790			0.988		
	Red	0.933			0.903		
	NIR	0.962			0.800		
IHS	Blue	0.830	0.837	9.283	0.924	0.872	5.132
	Green	0.772			0.947		
	Red	0.767			0.917		
	NIR	0.977			0.700		
IHSF	Blue	0.764	0.846	4.232	0.982	0.892	5.204
	Green	0.748			0.990		
	Red	0.881			0.978		
	NIR	0.991			0.619		
GS1	Blue	0.991	0.919	6.928	0.546	0.634	4.947
	Green	0.966			0.705		
	Red	0.990			0.376		
	NIR	0.729			0.910		
GSF	Blue	0.755	0.808	5.324	0.982	0.874	5.237
	Green	0.760			0.990		
	Red	0.745			0.981		
	NIR	0.972			0.544		
GS2	Blue	0.925	0.940	3.093	0.931	0.876	5.682
	Green	0.906			0.937		
	Red	0.935			0.934		
	NIR	0.993			0.702		
SFIM	Blue	0.869	0.930	2.986	0.940	0.869	5.630
	Green	0.909			0.914		
	Red	0.967			0.786		
	NIR	0.975			0.836		
HPF	Blue	0.923	0.948	2.655	0.933	0.844	5.570
	Green	0.913			0.930		
	Red	0.959			0.893		
	NIR	0.996			0.620		

**Table 4:** Pan-sharpening results achieved for frame 3.

Method	Bands	UIQI	UIQI_M_	ERGAS	ZI	ZI_M_	S-ERGAS
BT	Blue	0.793	0.828	7.271	0.963	0.946	6.134
	Green	0.840			0.973		
	Red	0.890			0.927		
	NIR	0.787			0.923		
BTF	Blue	0.762	0.832	5.969	0.974	0.936	7.015
	Green	0.830			0.987		
	Red	0.897			0.945		
	NIR	0.838			0.837		
IHS	Blue	0.812	0.835	7.859	0.954	0.929	8.120
	Green	0.786			0.970		
	Red	0.837			0.953		
	NIR	0.904			0.840		
IHSF	Blue	0.812	0.852	5.836	0.979	0.929	7.250
	Green	0.804			0.986		
	Red	0.872			0.972		
	NIR	0.920			0.777		
GS1	Blue	0.832	0.835	8.053	0.952	0.930	8.398
	Green	0.812			0.969		
	Red	0.789			0.963		
	NIR	0.905			0.838		
GSF	Blue	0.827	0.862	5.893	0.978	0.897	7.488
	Green	0.828			0.985		
	Red	0.832			0.982		
	NIR	0.962			0.641		
GS2	Blue	0.816	0.843	6.611	0.926	0.881	8.590
	Green	0.818			0.924		
	Red	0.818			0.932		
	NIR	0.919			0.740		
SFIM	Blue	0.765	0.821	6.407	0.934	0.881	8.628
	Green	0.827			0.902		
	Red	0.890			0.836		
	NIR	0.804			0.854		
HPF	Blue	0.844	0.874	5.429	0.908	0.856	8.289
	Green	0.840			0.909		
	Red	0.894			0.864		
	NIR	0.918			0.743		

**Table 5:** Pan-sharpening results achieved for Frame 4.

Method	Bands	UIQI	UIQI_M_	ERGAS	ZI	ZI_M_	S-ERGAS
BT	Blue	0.781	0.842	7.488	0.954	0.952	5.056
	Green	0.853			0.969		
	Red	0.892			0.948		
	NIR	0.842			0.935		
BTF	Blue	0.762	0.844	6.839	0.968	0.943	5.798
	Green	0.848			0.981		
	Red	0.896			0.953		
	NIR	0.868			0.872		
IHS	Blue	0.810	0.846	7.731	0.954	0.940	6.319
	Green	0.802			0.967		
	Red	0.858			0.962		
	NIR	0.913			0.878		
IHSF	Blue	0.817	0.860	6.650	0.975	0.938	6.100
	Green	0.820			0.982		
	Red	0.877			0.970		
	NIR	0.926			0.827		
GS1	Blue	0.856	0.843	7.960	0.953	0.954	6.242
	Green	0.834			0.966		
	Red	0.821			0.969		
	NIR	0.859			0.927		
GSF	Blue	0.851	0.863	6.756	0.974	0.946	6.176
	Green	0.844			0.981		
	Red	0.842			0.979		
	NIR	0.913			0.848		
GS2	Blue	0.826	0.826	8.042	0.915	0.905	13.206
	Green	0.821			0.914		
	Red	0.801			0.930		
	NIR	0.857			0.861		
SFIM	Blue	0.723	0.800	8.407	0.912	0.873	9.166
	Green	0.806			0.877		
	Red	0.856			0.853		
	NIR	0.817			0.850		
HPF	Blue	0.832	0.868	6.648	0.913	0.865	8.075
	Green	0.838			0.906		
	Red	0.883			0.872		
	NIR	0.921			0.771		

**Table 6:** Ranking of the pan-sharpening methods in each frame.

	Rural	Semi-Urban	Urban	Natural
**BT**	2	5	4	2
**BTF**	1	3	3	1
**IHS**	9	6	7	9
**IHSF**	5	1	2	4
**GS1**	7	7	5	6
**GSF**	8	2	1	8
**GS2**	4	8	8	7
**SFIM**	6	9	9	3
**HPF**	3	4	6	5

## Data Availability

The study’s data are available upon request from the corresponding authors for academic research and noncommercial purposes only. Restrictions apply to derivative images and models trained using the data, and proper referencing is required.
